# Energy absorption characteristics of E-glass/epoxy over-wrapped aluminum pipes with induced holes: an experimental research

**DOI:** 10.1038/s41598-022-25679-0

**Published:** 2022-12-06

**Authors:** Marwa A. Abd El-baky, Mahmoud M. Awd Allah, Madeha kamel, Walaa Abd-Elaziem

**Affiliations:** 1 Mechanical Design and Production Engineering Department, Zagazig University, Zagazig, 44519 Egypt; 2 Mechanical Engineering Department, Suez Canal University (SCU), Ismailia, Egypt

**Keywords:** Materials science, Engineering, Mechanical engineering

## Abstract

The effect of inducing circular holes into aluminum wrapped with glass/epoxy (Al/GFRP) pipes was investigated. Intact and holed specimens were evaluated in quasi-static axial compression after being constructed using the wet wrapping procedure. The effect of the induced holes' parameters i.e., hole diameter (d), number of holes (n), the hole position to specimen height ratio (L/H), on the crashworthiness of Al/GFRP structures was investigated. Results indicated that the existence of the cutouts visibly affects the values of crushing parameters. Increasing (d) remarkably reduces the total absorbed energy (U) and enhances the crushing force efficiency (CFE) of Al/GFRP pipes. Introducing two holes in two opposite faces with keeping their location constant reduces initial peak crush force ${(\mathrm{F}}_{\mathrm{ip}})$, mean crush force ${(\mathrm{F}}_{\mathrm{m}})$, and U of Al/GFRP pipes but increases CFE. As the hole location goes to the lower edge of the pipe i.e., L/H increases, U obviously increases. The specimens with 4 mm holes in one side at medium height and specimens with 12 mm holes on one side at L/H = 0.8 had the best crushing parameters, respectively. The adequate design of the induced cutouts in thin-walled constructions is valuable to enhance the crashworthy performance of engineering structures.

## Introduction

In recent decades, thin-walled structures are extensively used as energy dissipating devices in automobiles and aircrafts to improve crashworthiness^1–3^. The term “crashworthiness” can be defined as the ability of a material to protect its occupant from injuries during crush incidences^4,5^. In a number of technical fields, including as aviation, transportation, the nuclear industry, and civil engineering, lightweight structures with high energy absorption capacity are becoming more and more common^6–8^. Making a vehicle design as light as possible without sacrificing functional criteria like vehicle durability and safety is in high demand^9,10^.

The type of the material used in the manufacture of energy dissipating devices is a key factor that determines how efficient energy will be absorbed^11^. Traditionally, metals such as steel or aluminum have been utilized in crashworthy constructions as they display controllable plastic deformation, while polymeric composites (PCs) are fragile and cannot sustain this type of deformation^12^. PC structure has a substantially higher cost than standard metals due to the intricate design and fabrication process. On the other hand, PCs fail progressively which improves the energy absorption and they have lower density than metals, which enhances the specific properties and reduces the overall weight of the vehicle and consequently reduces the vehicle’s fuel consumption^13^. Also, if PCs are appropriately designed, they could gradually crash and delaminate to dissipate the impact energy^14–17^. PCs are characterized by their excellent specific stiffness and strength, outstanding energy absorbing capacity, non-conductivity, good corrosion resistance, and high thermal insulation^18–21^.

The advantages of increased specific strength and stiffness of FRP as well as the toughness of the metals can be combined to create hybrid structures out of metals and fiber reinforced polymers (FRP)^22^. Therefore, it is possible to create very desirable energy-absorbing structures by using the right amount of metallic components in combination with FRP composites^23^. Metal/FRP hybrid tubes' crushing reactions frequently result in plastic deformation for the metal component and a type of progressive crushing for the FRP component^24^. Metal/FRP hybrid structures have been extensively studied for their crashworthiness in the literature^25–28^.

For engineering specifications like internal connection and access or reducing the weight, introducing cutouts in energy dissipating devices walls is suggested^29^. Introducing cutouts offers two advantages. First, the initial peak force can be decreased and may be controlled. Second, such structures exhibit much less force oscillation during crushing^30^. The contributions of well-known researchers who studied the crashworthiness performance of metallic constructions included cutouts are displayed in Table 1.

**Table 1:** Summary of research works on crashworthiness performance of metallic structures with cutouts.

Reference	Material	Studied parameters	Loads	Study	Observation
Han et al.^31,32^	Steel and Al cylinders	Site of the cutouts	Quasi-static and dynamic impact	Numerical/experimental	When the cut-out site is over the mid-length, U of cylinder increases due to the progressive mode of crushing developed by the top end cutout
Shariati et al.^33^	Mild steel cylinders	Cutout position and cylinder parameters i.e., length/diameter ratio (L/D) and (b) diameter/thickness (D/t) ratio	Axial compression	Numerical/experimental	Changing the cutout location from mid-height to the edges of the cylinder increases the buckling load capacity. Cylinders with cutouts buckled locally, and then it exhibits general bending as the axial distortion progresses
Mamalis et al.^34^	Steel square tubes	Hole size and location	Quasi-static axial compression	Experimental	Existence of holes guarantees a stable collapse and reduces the initial peak force (${\mathrm{F}}_{\mathrm{ip}})$. The impact of the hole size on crushing response is less than its location. Specimens with a hole at middle height absorb larger amount of energy, reduce ${\mathrm{F}}_{\mathrm{ip}}$ and increase mean crush force (${\mathrm{F}}_{\mathrm{m}})$. Specimens with holes in one wall fail to decrease ${\mathrm{F}}_{\mathrm{ip}}$ and exhibit the same ${\mathrm{F}}_{\mathrm{m}}$ compared to specimens with holes at two opposite sides. The best behavior was recorded for specimens with 10 mm hole diameter in two opposite sides at the middle height of steel tubes
Bodlani et al.^35,36^	Mild steel square tubes	Circular holes with 17 mm diameter were drilled on two or four opposing tube sides of the tube to form opposing hole pairs. The total number of holes varies from 2 to 10	dynamic and quasi-static axial loads	Numerical/experimental	The existence of holes reduces ${\mathrm{F}}_{\mathrm{ip}} .$ Number of holes > 2 holes/side doesn’t considerably reduce ${\mathrm{F}}_{\mathrm{ip}}$. The existence of holes may affect U of the tube
Huang et al.^37^	Steel cylinders	Elliptical cutout locations, shapes, and symmetry	Axial impact	Numerical	Crushing behavior is greatly affected by the location and symmetry of cutouts and the variation of major axis affects ${\mathrm{F}}_{\mathrm{ip}}$
Taştan et al.^38^	Tapered Al-tubes	Diameter, and number of circular cutouts in horizontal and vertical directions	Quasi-static axial load	Numerical using Surrogate-based optimization approach	The optimum CFE and SEA of the holed tubes is 27.4 and 26.4% higher than those of the unholed tubes. Optimum SEA design has considerably increased cutout diameter, increased number of cutouts in horizontal direction and slightly decreased number of cutouts in vertical direction compared to the optimum CFE design
Sankar and Parameswaran^39^	Al-cylinders	Hole diameter and their spacing arrangement	Dynamic compression	Numerical/experimental	Hole diameter and their spacing arrangement have a great effect on ${\mathrm{F}}_{\mathrm{ip}}$ and on the deformation pattern. The existence of the holes considerably reduces ${\mathrm{F}}_{\mathrm{ip}}$. Holes localize the distortion to such an extent that the load at the formation of subsequent buckles increases, which negatively affects U. Larger number of smaller diameter holes caused higher ${\mathrm{F}}_{\mathrm{ip}}$ reduction
Baaskaran et al.^40^	Al-cylinders	Elliptical cut-outs’ location	Quasi-static axial load	Numerical/experimental	Cut-out’s location of greatly affects EAC and buckling characteristics of Al-tubes. The increase in the ellipse’s aspect ratio of the ellipse results in a decrease in ${\mathrm{F}}_{\mathrm{m}}$ which varies from 9.2 to 19.8%. Specimens with symmetrical cutout are much effective than those with single cutout
Nikkhah et al.^41^	Al-tubes	Shape of cutout	Axial/oblique loadings		Al-tubes with cutouts have lower ${\mathrm{F}}_{\mathrm{ip}}$ than perfect tube. ${\mathrm{F}}_{\mathrm{ip}}$ obviously decreases due to the existence of rectangular cutout. Al-tubes with circular and square cutouts have larger U than tubes with other cutouts at most load angles
Pirmohammad et al.^42^	Square and octagonal bi-tubal Al-structures	Hole shape and dimensions	Impact	Numerical	Hexagonal holes generated on square and octagonal bi-tubal Al-structures enhanced U by, respectively, 60 and 42% in comparison to the conventional structures. All holed structures displayed less ${\mathrm{F}}_{\mathrm{ip}}$ compared to those without holes. Square and octagonal structures with hexagonal holes are found as the best energy absorbing devices. The holes generated on the structure walls enhanced their EAC under oblique loading, as well
Patel et al.^43^	Al cap and open-end hybrid frusta	The existence of circular cut-outs	Quasi-static axial load	Numerical	CFE increased by 3–10%, through having a cut-out on both sets of hybrid frusta
Kathiresan^44^	Al-conical frusta	Cutout shape, location, and size	Quasi-static axial load	Numerical/experimental	Increasing the cutout size causes a decrease in ${\mathrm{F}}_{\mathrm{ip}}$ and ${\mathrm{F}}_{\mathrm{m}}$. Circular cutouts reveal better U than square or trapezoidal cutouts for conical frusta

FRP composites included cutouts have not been the subject of many investigations. Taheri-Behrooz et al.^45^ determined the axial force that E-glass/epoxy (GFRP) tubes with piercing can withstand. It was revealed that the unholed and holed tubes show the same instability mode profiles when subjected to axial force. But, the critical load and overall stiffness of the holed tubes were significantly reduced. The rigidity and load-carrying capability of composite tubes are negatively impacted by the presence of numerous tiny perforations. However, a moderate increase in the perforation's diameter and spacing has no appreciable impact on the outcome. For instance, as the perforation diameter increased from 2.5 to 15 mm, the tubes' axial rigidity reduced by 17 and 25%, respectively. Later on, an experimental research was done to determine how the hole diameter, vertical hole space, pipe diameter, hole pattern, transverse hole space, and the hole reinforcement affected the axial compressive behavior of perforated GFRP tubes, Wang et al.^46^. Perforated tubes' axial stiffness, critical load, and deformation capacity have all been shown to significantly decrease. The perforation pattern, transverse hole spacing, hole diameter, and tube diameter all had a big impact on how the tubes behaved when compressed axially. The impacts of vertical hole spacing and hole reinforcement, however, were barely noticeable.

Through the use of quasi-static axial compression, Kathiresan et al.^47^ investigated how the addition of circular, square, and elliptical cuts altered the U and deformation features of conical frusta made of GFRP. Additionally, conical frusta crush behavior was simulated using finite element software, and the resulting results were compared to the experimental ones. According to Zbek et al.'s^48^ investigation into the impact of adding a circular cut-out trigger mechanism on GFRP tubes made using the filament wrapping technology under quasi-static axial load, the performance of circular cut-outs can be improved in terms of crashworthiness.

Liu et al.^49^ investigated the impact of holes’ size, figure and spreading on both the bearing performance and the failure signs of tubes made of carbon fiber-reinforced plastic (CFRP). Compared with the intact CFRP specimens, the holed tubes’ ${\mathrm{F}}_{\mathrm{ip}}$ and SEA were decreased by, respectively, 3–22% and 26–57%. The holes’ size effect on the tube failure strength is less profound than that of the laminates but compared to the distribution and shape of the holes, it is considerably stronger. Alhyari and Newaz^50^ calculated the U of CFRP tubes that were subjected to quasi-static stresses and had single or double induced holes in specific locations with varying diameters. With single or double perforations that are 15 mm in diameter and 100 mm from the top of the tube, SEA has been seen to be reduced by 50%. When the hole is 20 mm in diameter and 100 mm from the top of the tube, the SEA drop is about 60% less than it would be for an undamaged tube. When the holes are 75 mm from the top of the tube, the reduction is the greatest. The hole’s location (from 100 to 75 mm) can generate more pronounced influence than the size of the hole (15 vs. 20 mm) for the studied specimens.

Numerous studies have been done metal/polymer structures to merge the high specific strength and stiffness of composites with the plastic deformation of metals^51–53^. But few works investigate the effect of introduced cut-outs on hybrid structure, Shun et al.^1^ explored how the induced holes affect the crushing behavior of aluminum (Al)/CFRP members studied under quasi-static axial loads. The dimension and number of holes have a significant impact on the crushing behaviour, according to the results. The parameters of the induced holes were optimized using multi-objective optimization. Compared with the intact Al/CFRP tube, ${\mathrm{F}}_{\mathrm{ip}}$ of the optimum specimen reduced by 24.32% and SEA is slightly enriched by 0.68%. According to the research done by Alshahrani et al.^18^, the criteria that can effectively regulate the performance of crashworthiness and alter the failure mechanism of Al/polymer composite cylinders include the diameter, the number of induced holes, and the number of fiber/epoxy layers.

Recently, it was observed that metal-PC hybrid architectures are adapted in the automotive industry because of their excellent versatility and crashworthiness. However, very few studies have concentrated on illuminating the mechanisms through which metal-PC hybrids absorb energy. Moreover, it is clear from the review above that few experiments were done to look at how cutouts affect the performance of metal/PC hybrid pipes and it is clear that more research efforts should be intensified towards the windowed hybrid metal-PC pipes. Therefore, the primary goal of the current study is to experimentally investigate how well Al/GFRP thin-walled tubular constructions with circular cutouts perform in terms of crashworthiness. Wet wrapping was used to create the specimens, which were then put through testing with nearly static axial loads. The hole diameter (d = 0, 4, 8, 12 mm), hole position to height ratio (L/H = 0.5, 0.6, 0.7, 0.8), and number of holes (n = 1, 2) are the research factors. To comprehend the impacts of circular cuts on the energy absorption and fracture characteristics of GFRP over wrapped Al pipe samples, the crush characteristics of these three groups were investigated.

## Methodology

According to numerous authors^14,29^, uniaxial quasi-static crush tests do not currently have a standard. Crosshead speed of 10 mm/min was adapted in uniaxial quasi-static crush tests based on many studies in the literature^18,54–56^. Since circular samples absorb more energy and reduce stress concentrations than square or rectangular samples, they were employed to facilitate crushing testing^57^. The studied variables are the hole diameter (d = 4, 8, 12 mm), the hole position to height ratio (L/H = 0.5, 0.6, 0.7, 0.8), the number of holes (n = 0, 1, 2).

### Materials

As a reinforcement, a woven E-glass fabric (200 g/m^2^) supplied by Hebei Yuniu Fiber Glass Manufacturing Co. Ltd. China was used. Chemicals for Modern Buildings Co. Ltd. Egypt supplied Kemapoxy 150RGL matrix. The mechanical characteristics of E-glass and Kemapoxy 150 RGL are shown in Table 2. Al6063 aluminum alloy pipe with 50 mm outer diameter and 2 mm thickness was given by Military Production Co. Ltd. Egypt. Table 3 lists the chemical make-up of Al6063 in weight percentages.

**Table 2:** Properties of reinforcement and matrix, given by the supplier.

Property	Reinforcement	Matrix
Density, g/cm^3^	2.5	1.1
Young’s modulus, GPa	76	1.2
Poisson’s ratio	0.22	0.35
Elongation, %	1.8–3.2	2.2–2.9
Tensile strength, MPa	3400	55–58

**Table 3:** Chemical composition of Al6063 (wt%).

Si	Fe	Cu	Mn	Mg	Zn	Ti	Al
0.44	0.18	0.01	0.04	0.48	0.01	0.01	Bal

### Surface treatment of Al-pipes

**Figure 1:**
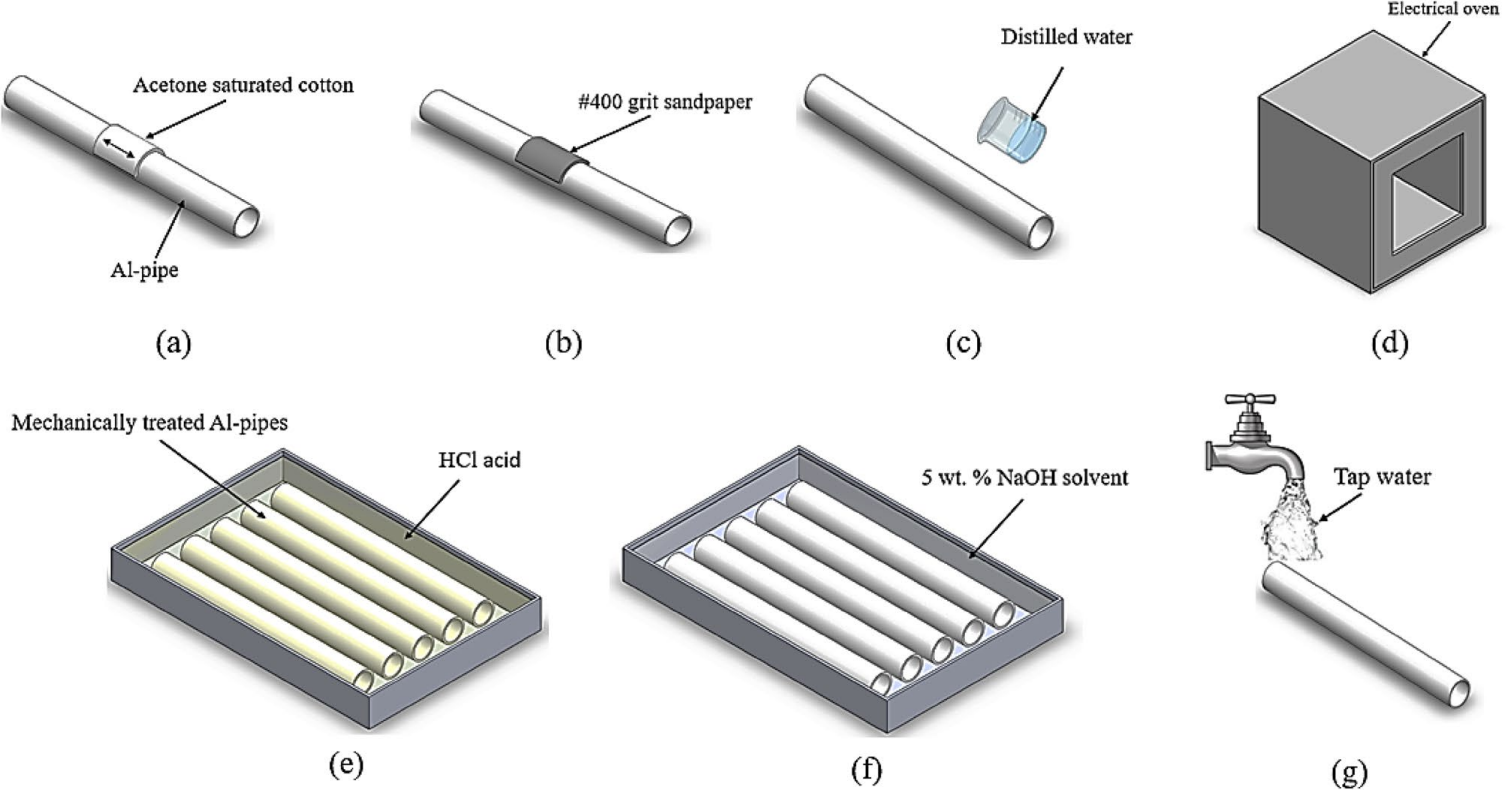
Surface treatment of Al-pipes.

Al-alloy pipes were subjected to mechanical and chemical treatments to ensure a strong connection with GFRP, see Fig. 1. Al-pipes underwent mechanical treatment by being rinsed with acetone, Fig. 1a. Then pipes were smoothly abraded with #400-grit sandpaper Fig. 1b, after that the pipes were rinsed with distilled water, Fig. 1c. Finally, the pipes were dried in an oven, Fig. 1d. To increase the surface roughness of the mechanically treated Al-pipes, a chemical treatment utilizing acid washing with HCl having an 11% volumetric concentration was applied, Fig. 1e. For 30 min, acid etching was done at room temperature. Al-pipes were dried after being rinsed with distilled water. Al-pipes were then submerged in a 5 weight percent NaOH solution at 70 °C for 5 min, see Fig. 1f. The oxidized Al-pipes were rinsed with tap water to remove the oxide residue after being baked in an oven to stabilize the oxide layer, as shown in Fig. 1g ^58–60^.

### Fabrication of Al/GFRP specimens

**Figure 2:**
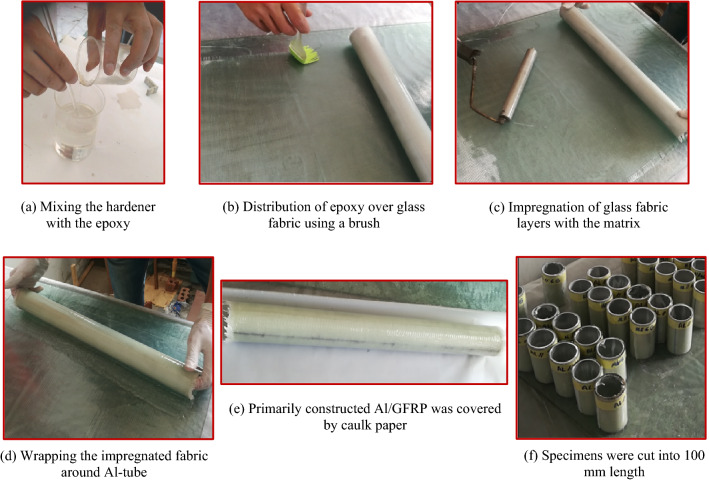
Steps of fabricating Al/GFRP composite tubes.

**Figure 3:**
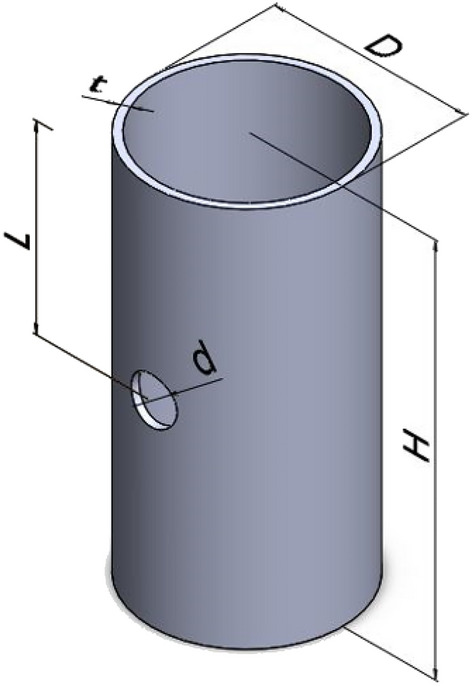
A schematic view of test specimens.

In this study, Al/GFRP hybrid pipes were constructed using a wet-wrapping procedure done by hand. The steps of the fabrication process are as follows:

- For 5 min, epoxy and its hardener were manually combined and swirled, Fig. 2a. Using a brush, the liquid was then evenly applied to the glass cloth, Fig. 2b. Additionally, glass fabric layers were impregnated with the matrix using a metallic roller, Fig. 2c.
- 50 mm-diameter and 2 mm-thick Al pipes were wrapped with the impregnated cloth, Fig. 2d. Each Al-pipes was wrapped by 8 glass/epoxy plies.
- The constructed Al/GFRP hybrid pipes were shielded by caulk paper and let in room temperature for seven days to be cured, Fig. 2e. According to the data given by the supplier, curing conditions of Kemapoxy 150 RGL are 8 h initial setting time, 24 h final setting time, and 7 days’ full hardness time. Attia et al.^61^ and Awd Allah et al.^62^ adapted the above-mentioned curing conditions.
- The manufactured hybrid pipes were visually examined for material flaws and geometrical abnormalities after curing. The samples were separated into 100 mm-long pieces. The chosen holes were made using a tungsten carbide twist drill, Fig. 2f. A schematic of test specimens is shown in Fig. 3. Table 4 provides a list of the test specimens' geometrical dimensions.

**Table 4:** Description of the fabricated Al/GFRP hybrid tubes with induced holes.

Code	Description	Diameter, d (mm)	Number of holes, n	Height, H (mm)	Mass, M (g)	Hole position to specimen height ratio, L/H	${\updelta }_{\mathrm{max}}$, (mm)
A0	Al cylinder over-wrapped with eight layers of glass reinforced epoxy	–	–	96.00	125.50	–	71.02
d4-n1-r0.5		4	1	97.14	124.60	0.5	67.63
d8-n1-r0.5		8	1	100.25	127.66	0.5	59.93
d12-n1-r0.5		12	1	98.53	125.38	0.5	58.97
d4-n2-r0.5		4	2	99.00	126.50	0.5	66.56
d8-n2-r0.5		8	2	95.65	120.00	0.5	64.05
d12-n2-r0.5		12	2	97.50	123.46	0.5	60.79
d12-n1-r0.6		12	1	101.05	125.29	0.6	60.27
d12-n1-r0.7		12	1	96.05	123.49	0.7	61.59
d12-n1-r0.8		12	1	99.75	128.64	0.8	62.59

The letters “d”, “n” and “r” in the specimen code represent the diameter and number of the holes and position to height ratio, respectively. For examples, “A0” means Al-cylinder over-wrapped with eight glass/epoxy layers with no holes. d4-n2-r0.5 means Al-cylinder over-wrapped with eight glass/epoxy layers with two opposite holes with 4 mm diameter and 0.5-hole position to height ratio.

### Testing

**Figure 4:**
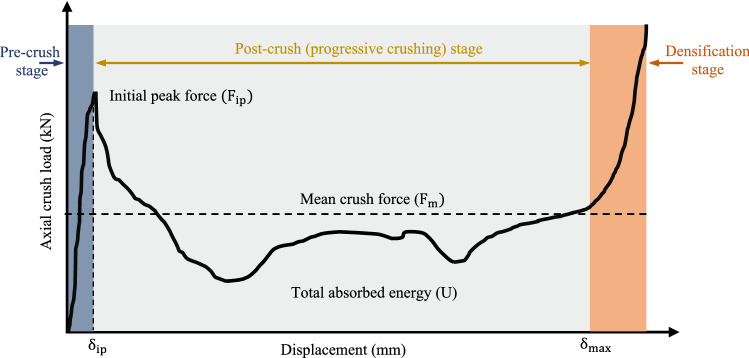
Typical load–displacement curve of composite energy absorption tube.

Quasi-static axial compression is a recognized test to study the energy absorption capacity of polymer composites^6^. It was performed on Al/GFRP specimens with induced holes using a universal testing machine with 100 kN capacity. Load–displacement data was directly recorded using an automatic data acquisition system. Additionally, the histories of deformation for test specimens were tracked and documented. For each case, three samples were examined, and the average was then given. The generated load versus displacement plots can be used to objectively assess the performance of crashworthy Al/GFRP specimens. Figure 4 schematically demonstrates the typical load–displacement plot attained from axial crushing test. The crushing key parameters were determined as follows: -

- Initial peak force (${\mathrm{F}}_{\mathrm{ip}})$ can be directly recorded from the crushing load versus displacement plot. It is recommended to be small enough to avoid the transformation of the crushing force from the energy absorber to the main vehicle body.
- Total absorbed energy (U) can be calculated as follows:
  1 $$\mathrm{U}= {\int }_{0}^{{\updelta }_{\mathrm{max}}}\mathrm{ F}\left(\updelta \right)\mathrm{ d\delta },$$
  where, $\mathrm{F}\left(\updelta \right)\mathrm{ and }{\delta }_{\mathrm{max}}$ are the instant load and overall displacement, respectively.
- Mean crush force (${\mathrm{F}}_{\mathrm{m}})$ can be obtained from the following equation:
  2 $${\mathrm{F}}_{\mathrm{m}}= \frac{{\int }_{0}^{{\updelta }_{\mathrm{max}}}\mathrm{ F}\left(\updelta \right)\mathrm{ d\delta }}{{\delta }_{\mathrm{max}}}.$$
- Crushing force efficiency (CFE) can be calculated as follows:
  3 $$\mathrm{CFE}= \frac{{\mathrm{F}}_{\mathrm{m}}}{{\mathrm{F}}_{\mathrm{ip}}} \times 100.$$
- Specific absorbed energy (SEA) is (U) divided by the mass of the component ($\mathrm{M}$):
  4 $$\mathrm{SEA}=\frac{\mathrm{U}}{\mathrm{M}}.$$

## Results and discussions

### Hole diameter (d) effect

**Figure 5:**
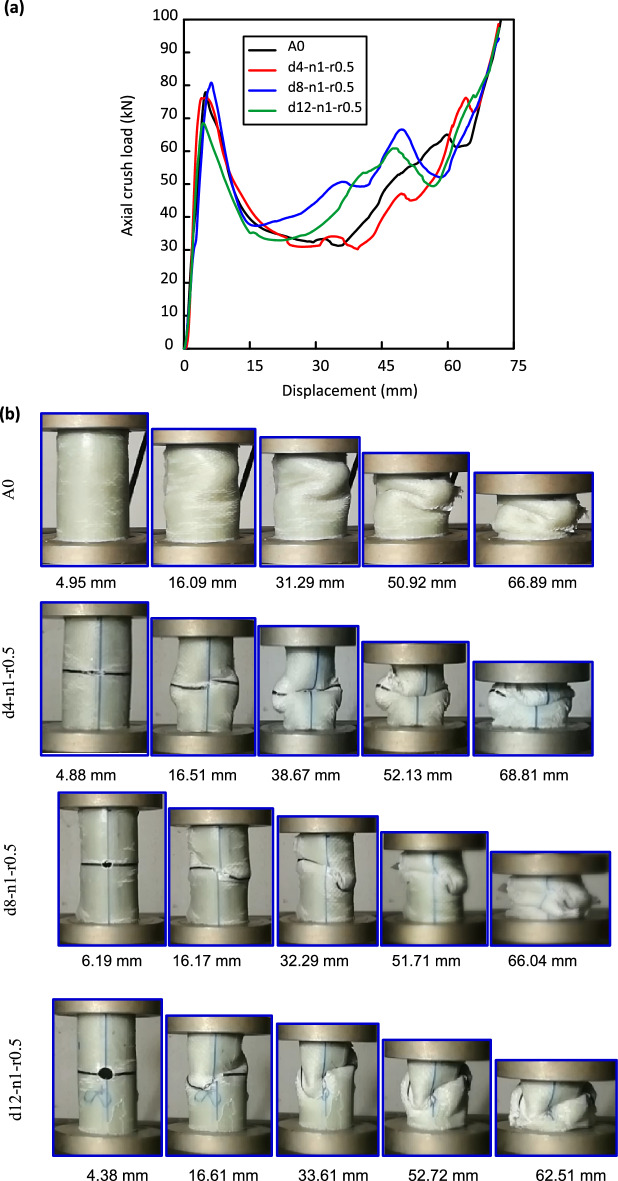
Al/GFRP specimens with different holes’ diameter (d = 0, 4, 8 and 12 mm) (**a**) load–displacement curves, and (**b**) crushing history.

Figure 5 displays the force versus displacement plots and the damage history for Al/GFRP samples with induced holes. Holes with diameters of 0, 4, 8, and 12 mm were employed at the specimens’ mid length to study the influence of the hole diameter on crashworthiness performance of E-glass/epoxy over-wrapped Al-pipes. As illustrated in Fig. 5a, A0, d4-n1-r0.5, d8-n1-r0.5, and d12-n1-r0.5 specimens behave linear till they reach ${\mathrm{F}}_{\mathrm{ip}}$ of 77.91 kN, 76.09 kN, 80.80 kN and 68.51 kN, respectively. A sharp load drop was noticed after ${\mathrm{F}}_{\mathrm{ip}}$ due to crack initiation and propagation. Intact and perforated pipes have the same instability mode shapes in the post crushing zone. Global buckling accompanied with matrix cracking, delamination, and fiber breakage in the intact specimen wall can be observed, Fig. 5b. For perforated pipes, cracks start close to the hole and propagate in the peripheral direction with noticeable wrinkling. Holed tubes prone to collapse in the mid-height. This outcome aligns with that attained by Liu et al.^63^ for CFRP tubes.

It is clear from Table 5 that a slight decrease in ${\mathrm{F}}_{\mathrm{ip}}$ of Al/GFRP composite pipes was noticed by inducing a center hole with 4 mm diameter. 12.07% decrease in ${\mathrm{F}}_{\mathrm{ip}}$ of Al/GFRP composite pipes was noticed by inducing a enter hole with 12 mm diameter. As reported by Awd Allah et al.^29^, it is advised to include cutouts to the walls of crashworthy components for technical purposes such as connecting and weight reduction. A key design objective is to reduce the overall vehicle weight. Fuel consumption will decrease when vehicle weight decreases. The high strength to weight ratio, which is directly associated to fuel consumption and reduction of CO_2_ emissions, is an important characteristic in designing of transportation systems^64^. The introduction of any discontinuities along the walls of crashworthy components allows for a decrease in the peak load^65^. As reports by Guler et al.^66^, initial peak load should be low enough to avoid the transformation of the forces from the crush element to the main body of vehicle. It can be seen that perforating the tubes with holes of d = 4 mm decreases the peak loads a little. A fluctuation in peak load values for holed specimens was recorded. Rouzegar et al.^67^ and liu et al.^63^ recorded similar results for the hesitation in peak load values of Al over wrapped E-glass/vinyl ester tubes. Song et al.^68^ recorded the same trend in peak load values. The reason is mainly due to the initial overall stiffness of the tubes after the introduction of cutouts.

**Table 5:** Crashworthiness parameters for Al/GFRP thin-walled circular tubes with induced holes.

Specimen code	Calculations	${\mathrm{F}}_{\mathrm{ip}}$, kN	U, J	SEA, J/g	${\mathrm{F}}_{\mathrm{m}}$, kN	CFE
A0	Avg	77.91	3441.14	27.42	48.45	0.62
	SD	6.38	249.59	3.24	4.59	0.03
	CV, %	8.19	7.25	11.82	9.47	4.84
d4-n1-r0.5	Avg	76.09	3058.63	24.55	45.23	0.59
	SD	4.42	350.32	3.07	3.53	0.06
	CV, %	5.81	11.45	12.51	7.80	10.85
d8-n1-r0.5	Avg	80.82	2967.21	23.24	49.51	0.61
	SD	8.78	312.46	2.19	3.19	0.02
	CV, %	10.86	10.53	9.42	6.44	3.28
d12-n1-r0.5	Avg	68.51	2677.44	21.35	45.40	0.66
	SD	6.12	281.43	1.42	3.78	0.05
	CV, %	8.93	10.51	6.65	8.33	8.03
d4-n2-r0.5	Avg	71.40	3009.47	23.79	45.21	0.63
	SD	5.85	279.96	2.51	2.94	0.09
	CV, %	8.19	9.30	10.55	6.50	14.76
d8-n2-r0.5	Avg	73.07	2881.04	24.01	44.98	0.62
	SD	6.82	375.06	2.22	3.66	0.03
	CV, %	9.33	13.02	9.25	8.14	4.52
d12-n2-r0.5	Avg	62.39	2548.77	20.64	41.93	0.67
	SD	7.26	198.64	1.98	2.50	0.07
	CV, %	11.64	7.79	9.59	5.96	10.45
d12-n1-r0.6	Avg	70.08	2832.19	22.61	46.99	0.67
	SD	6.58	276.71	3.24	3.98	0.08
	CV, %	9.39	9.77	14.33	8.47	11.94
d12-n1-r0.7	Avg	75.00	2848.21	23.06	46.24	0.62
	SD	5.63	337.60	1.83	3.11	0.04
	CV, %	7.51	11.85	7.94	6.73	6.45
d12-n1-r0.8	Avg	70.03	2861.25	22.24	45.71	0.65
	SD	5.17	356.87	3.17	3.53	0.09
	CV, %	7.38	12.47	14.25	7.72	13.85

A0 specimen exhibits the maximum U with a value of 3441.14 J followed by d4-n1-r0.5 with a value of 3058.63 J. Presence of small holes in Al/GFRP pipes adversely affects their U and SEA. Increasing the diameter of the hole from 4 to 12 mm decreases SEA of the pipe by 11.11 and 22.14%. The existence of cutouts has a negative effect on U of square CFRP tubes according to the research conducted by Liu et al.^63^. As reported by Fan et al.^69^, when choosing cylinders as energy absorbing elements, it is significant that cylinders of high SEA are chosen, to maximize the energy absorption while keeping the structure light. d12-n1-r0.5 specimen exhibits the higher CFE with a value of 0.66. The obtained results is consistent with that obtained by Alhyari and Newaz^50^ for CFRP tubes.

### Number of holes (n) effect

**Figure 6:**
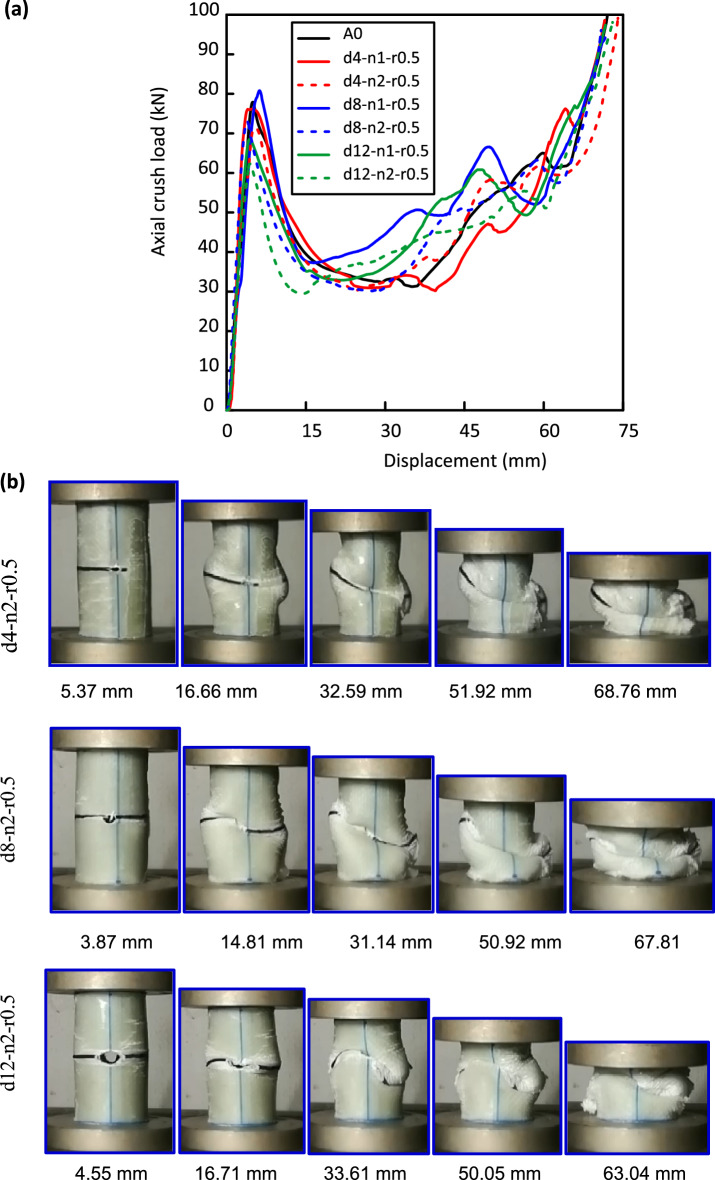
Al/GFRP specimens with different numbers of holes, (**a**) load–displacement curves and (**b**) crushing history.

After the examination of hole diameter (d) effect, the next step is to analyze of the effect of the number of holes (n) on the crashworthiness. Al/GFRP samples with three numbers of holes (0, 1, and 2) at the specimen mid-length were used to investigate the impact of cutout number on crush performance. Figure 6 shows the load versus displacement plots and damage history for different samples with the selected hole numbers. All specimens have linear trends till they approach ${\mathrm{F}}_{\mathrm{ip}}$ values at 71.4 kN, 73.07 kN and 62.39 kN for d4-n2-r0.5, d8-n2-r0.5 and d12-n2-r0.5 specimens, respectively. Then a sharp decrease in the load after ${\mathrm{F}}_{\mathrm{ip}}$ was detected due to crack propagation and folds that appear in the vicinity of the hole. Obvious fluctuations were recorded in the post crushing zones for all specimens.

It is clear from Table 5 that ${\mathrm{F}}_{\mathrm{ip}}$ values of d4-n2-r0.5, d8-n2-r0.5 and d12-n2-r0.5 specimens decrease, respectively, by 8.36, 6.25, and 19.92% compared with A0 specimen. ${\mathrm{F}}_{\mathrm{ip}}$ values of d4-n2-r0.5, d8-n2-r0.5 and d12-n2-r0.5 specimens decrease, respectively, by 6.16, 9.59, and 8.93% compared, respectively, with d4-n1-r0.5, d8-n1-r0.5 and d12-n1-r0.5 specimens. U values of double hole specimens are lower than those of single hole specimens. The reduction in ${\mathrm{F}}_{\mathrm{ip}}$ value is owing to cracks generated around the cut-outs and propagated due to the high-stress concentrations. These cracks weaken the specimens causing a decrease in ${\mathrm{F}}_{\mathrm{ip}}$ value. This result agrees with that obtained by Özbek et al.^48^.

$U$ values of d4-n2-r0.5, d8-n2-r0.5 and d12-n2-r0.5 specimens decrease, respectively, by 12.54, 16.28, and 25.93% compared with A0 specimen. $\mathrm{U}$ values of d4-n2-r0.5, d8-n2-r0.5 and d12-n2-r0.5 specimens decrease, respectively, by 1.61, 2.9, and 4.81% % compared, respectively, with d4-n1-r0.5, d8-n1-r0.5 and d12-n1-r0.5 specimens. Results are in line with what was discovered by Shun et al.^1^ for Al/CFRP thin-walled structures. Max CFE with a value of 0.67 was observed for d12-n2-r0.5 specimen against a value of 0.66 for d12-n1-r0.5 specimen. This means that presenting two holes instead of one hole in a known place has approximately no effect on CFE.

### Hole position to specimen height ratio (L/H) effect

**Figure 7:**
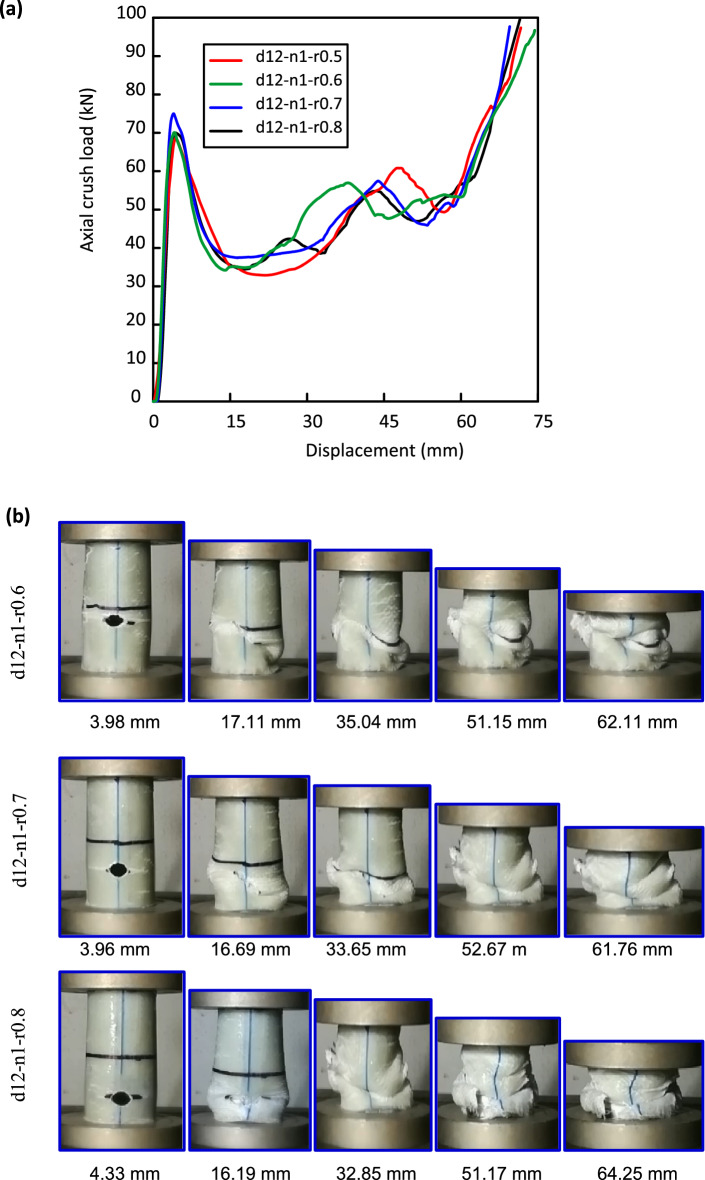
Al/GFRP specimens with different hole locations (**a**) load–displacement curves and (**b**) crushing history.

In this section of the study, the effect of the hole location on crashworthiness performance of Al/GFRP pipes was examined. Figure 7 displays the load versus displacement plots and damage history for Al/GFRP samples with 12 mm hole size where the hole position to height ratio (L/H) is 0.5, 0.6, 0.7, and 0.8. d12-n1-r0.6, d12-n1-r0.7 and d12-n1-r0.8 specimens perform linear till they reach ${\mathrm{F}}_{\mathrm{ip}}$ at 70.08, 75.00, and 70.03 kN, respectively. The reason is that the extra increase in “the hole position to height ratio” can decrease the remaining material at the bottom edge of the holed specimens and consequently decreases the specimen stiffness^68^. Later ${\mathrm{F}}_{\mathrm{ip}}$ sharply dropped accompanied with oscillations which reflects the unstable behavior of the pipes (Fig. 7a). Cracks around the holes accompanied with buckling and wrinkling of the specimens’ wall were recorded (Fig. 7b).

Compared with A0 specimen, ${\mathrm{F}}_{\mathrm{ip}}$ of d12-n1-r0.6, d12-n1-r0.7 and d12-n1-r0.8 specimens decreases by 10.05, 3.74, and 10.11%, respectively. Max CFE with a value of 0.67 was detected for d12-n1-r0.6 specimen against a value of 0.66 for d12-n1-r0.5 specimen. It can be concluded that (L/H) of the cut-out has no weighty effect on CFE. Improving in U was observed by increasing (L/H). Against d12-n1-r0.5 specimen, an improvement of 5.78, 6.38, and 6.87% in U was recorded for d12-n1-r0.6, d12-n1-r0.7 and d12-n1-r0.8 specimens, respectively.

### Scatter in the obtained results

The manufacturing, preparation, handling, storage, test rig design, and experimental technique of the specimens are just a few of the many variables that can influence the test findings. The experimental test findings' statistical scatter, or coefficient of variation (CV), has been determined and is shown in Table 5. The maximum CV for ${\mathrm{F}}_{\mathrm{ip}}$, U, SEA, ${\mathrm{F}}_{\mathrm{m}}$, and CFE was noted to be 11.64% for d12-n2-r0.5, 13.02% for d8-n2-r0.5, 14.33% for d12-n1-r0.6, 9.47% for A0, and 14.76% for d4-n2-r0.5, respectively. It is clear that the coefficient of variation (CV) values of all results are less than 15%, which confirm the repeatability of the results and reflect noticeable accuracy. Rouzegar et al.^67^ attributed the deviations observed between the results to the fabrication imperfections.

### Mechanisms of failure

**Figure 8:**
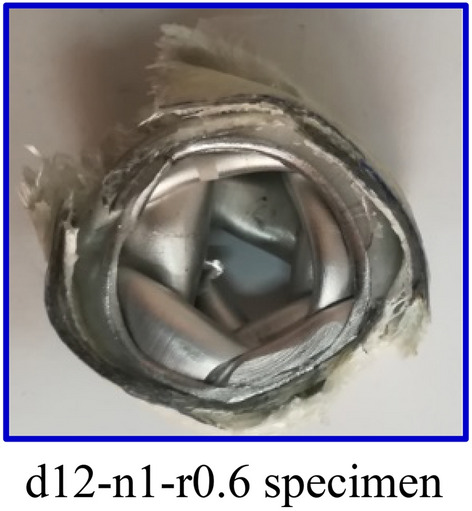
Top view of the representative sample of crushed specimens.

An important factor to be considered when examining the energy-absorbing capability of Al/GFRP pipes with cuts is the failure mechanism. The same failure mode was observed for intact and perforated pipes. Pipes first buckle, and then macro cracks in the matrix start to form around the holes. The cracks then spread outward in the direction of the pipes' periphery. As the cracks spread further, the lamina bends and forms internal and exterior folds. Delamination, fiber breakage and fiber pull-out can be noticed around the holes accompanied with global buckling and wrinkling. These signs of failure were recorded by Mache et al.^70^ for jute/polyester and glass/polyester tubes. Figure 8 demonstrates top view of the representative sample of crushed specimens.

## Applications

**Figure 9:**
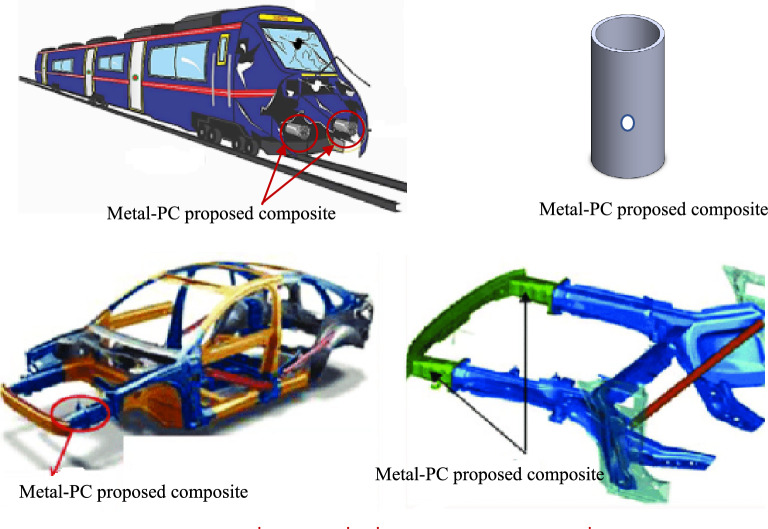
Applications of the proposed hybrids.

The suggested metal-PC hybrid composites can be employed in high-performance applications and safety equipment in the transportation sectors including the maritime, aerospace, and automobile industries. The proposed pipes can be used as energy-absorbing parts in forward-facing vehicle structures i.e., impact-resistant rods or crush boxes, see Fig. 9. In addition, they can be adapted to be used in the fuselage of aircrafts.

## Future trend

Future research will examine patterns with various window shapes and arrangements. The proposed window method can be expanded to pipes with varied cross-sectional forms and different loading situations due to its simplicity compared to other pattern design methods, which will be investigated in later studies.

## Conclusions

In this paper, it was suggested to use circular tubes made of aluminum (Al) and glass fiber-reinforced polymer (GFRP) with cuts in the shape of circular holes as crush boxes for automobiles. To investigate the impact of the induced holes on the crashworthiness performances, the effect of hole diameter (d), hole number (n), and hole position to specimen height ratio (L/H) were explored. The inferences that can be made are as follows:

- The crashworthiness performance and failure mechanism of Al/GFRP circular pipes exposed to quasi-static axial crushing were found to be greatly influenced by the hole variables, namely hole diameter (d), hole number (n), and the hole position to specimen height ratio (L/H).
- Increasing “d” decreases the initial peak crush force ${(\mathrm{F}}_{\mathrm{ip}})$, overall absorbed energy (U), and specific absorbed energy (SAE) but enhances the crushing force efficiency (CFE) of Al/GFRP pipes.
- When n = 2 instead of 1, this reduces ${(\mathrm{F}}_{\mathrm{ip}})$ and (U), but slightly enhances (CFE) of Al/GFRP pipes.
- Increasing (L/H) from 0.5 to 0.8, increases ${(\mathrm{F}}_{\mathrm{ip}})$ and (U) with approximately no effect on CFE of Al/GFRP pipes.
- Adequate design of induced holes in thin-walled structure is important to enhance the crashworthy performance of engineering structures.

## Data Availability

The datasets generated during and/or analyzed during the current study are available from the corresponding author on reasonable request.
